# A resource for whole-body gene expression map of human tissues based on integration of single cell and bulk transcriptomics

**DOI:** 10.1186/s13059-025-03616-4

**Published:** 2025-06-03

**Authors:** Mengnan Shi, Loren Méar, Max Karlsson, María Bueno Álvez, Andreas Digre, Rutger Schutten, Borbala Katona, Jimmy Vuu, Emil Lindström, Feria Hikmet, Han Jin, Meng Yuan, Xiangyu Li, Hong Yang, Xiya Song, Evelina Sjöstedt, Fredrik Edfors, Per Oksvold, Kalle von Feilitzen, Martin Zwahlen, Mattias Forsberg, Fredric Johansson, Jan Mulder, Tomas Hökfelt, Yonglun Luo, Lynn Butler, Wen Zhong, Adil Mardinoglu, Åsa Sivertsson, Fredrik Ponten, Linn Fagerberg, Cecilia Lindskog, Mathias Uhlén, Cheng Zhang

**Affiliations:** 1 Science for Life Laboratory, Department of Protein Science, KTH-Royal Institute of Technology, Stockholm, Sweden; 2 Department of Immunology, Genetics and Pathology, Uppsala University, Uppsala, Sweden; 3 Division of Obstetrics and Gynecology, Department of Clinical Science, Intervention and Technology, Karolinska Institutet, Stockholm, 14186 Sweden; 4 Department of Molecular and Cellular Biology, Center for Brain Science, Harvard University, Cambridge, MA USA; 5 Department of Neuroscience, Karolinska Institutet, Stockholm, Sweden; 6 Steno Diabetes Center Aarhus, Aarhus University Hospital, Aarhus, Denmark; 7 Department of Biomedicine, Aarhus University, Aarhus, Denmark; 8 Department of Clinical Medicine, The Arctic University of Norway, Tromsø, Norway; 9 Clinical Chemistry and Blood Coagulation Research, Department of Molecular Medicine and Surgery, Karolinska Institute, Stockholm, Sweden; 10 Science for Life Laboratory, Department of Biomedical and Clinical Sciences (BKV), Linköping University, Linköping, Sweden

**Keywords:** Single-cell, Gene expression mapping, Cell type classification, Human Protein Atlas

## Abstract

New technologies enable single-cell transcriptome analysis, mapping genome-wide expression across the human body. Here, we present an extended analysis of protein-coding genes in all major human tissues and organs, combining single-cell and bulk transcriptomics. To enhance transcriptome depth, 31 tissues were analyzed using a pooling method, identifying 557 unique cell clusters, manually annotated by marker gene expression. Genes were classified by body-wide expression and validated through antibody-based profiling. All results are available in the updated open-access Single Cell Type section of the Human Protein Atlas for genome-wide exploration of genes, proteins, and their spatial distribution in cells.

**Supplementary Information:**

The online version contains supplementary material available at 10.1186/s13059-025-03616-4.

## Background

The rapid advancement of omics technologies has enabled large-scale projects to map gene expression across all major human tissues and organs. Projects like the Human Cell Atlas (www.humancellatlas.org) [1] and the Tabula Sapiens [2] aim to systematically profile every cell type using single-cell sequencing, while specialized initiatives such as the Allen Brain Atlas [3, 4] focus on specific tissues using both single-cell and spatial transcriptomics. These projects have offered whole-genome transcriptomic measurements of healthy tissues, empowering the exploration of cell type-specific gene expression across the majority of human tissues and organs.

However, single cell analysis comes with some challenges. This includes issues with the generation of cells for some tissues, in particular the brain, and the low coverage of genes due to relatively few sequencing reads coming from each single cell. To overcome these difficulties, we previously adopted a tissue “pooling” strategy [5] to increase the depth and breadth of the single cell analysis. The analysis combined data from individual cell clusters to normalize the expression of all protein-coding genes, thereby defining both cell type-specific and housekeeping genes at the level of individual cell types [5]. This approach was further enhanced by high-resolution antibody-based profiling, providing visual representations of single cells within intact tissue samples for the inaugural version of the Single Cell Type Atlas within the HPA.

Complementary to single-cell transcriptomics, bulk RNA sequencing remains a powerful tool, offering extensive coverage across all the protein-coding genes of the human genome and thus allows also low abundant genes to be explored. Notable amongst publicly available bulk transcriptomics efforts are the Genotype-Tissue Expression (GTEx) project [6] and the Human Protein Atlas (HPA) [6]. These comprehensive ventures have delineated two principal gene categories: housekeeping genes, indispensable for universal cellular functions, and tissue-specific genes, responsible for unique, tissue-dependent functionalities. The examination of tissue-specific genes sheds light on the distinct biological processes unfolding within different tissues, offering critical insights into tissue development, function, and disease target [7–9].

However, bulk transcriptomics, despite its merits, comes with a significant drawback. It provides an average gene expression across a myriad of cells in a tissue sample, hence lacking the resolution to detect variations in gene expression between different cell types within the same tissue [10, 11]. This leads to potential oversight of genes that are expressed in specific cell types, since their signal can be diluted or masked by the amalgamated expression data, particularly when those cell types, such as immune cells and fibroblasts, are ubiquitous across various tissues [12, 13].

In this report, we present an expanded genome-wide resource of protein-coding genes involving major human tissues and organs using single-cell transcriptomics, complemented by bulk transcriptomics. A full-body cell taxonomy analysis has now been performed on 31 tissues using the previously described [5] pooling method, leading to refined classifications of genes based on their cell type specificity. Comparative bulk transcriptomic analysis of the corresponding tissues was also conducted to validate the data and systematically compare cell and tissue-specific transcripts. All of the results have been added to the updated open access resource on the Human Protein Atlas (www.proteinatlas.org) under Single Cell Type section. The genes expressed in each of the cell types can be explored in interactive UMAP plots and bar charts, with links to corresponding immunohistochemical stainings in human tissues. Additionally, these data are all downloadable via the HPA, which is suitable as input for advanced single cell data analysis including AI-based efforts to model the expression in single cells across the different tissues and organs of the human body.

## Construction and content

### The transcriptional landscape of single cell types

**Fig. 1:**
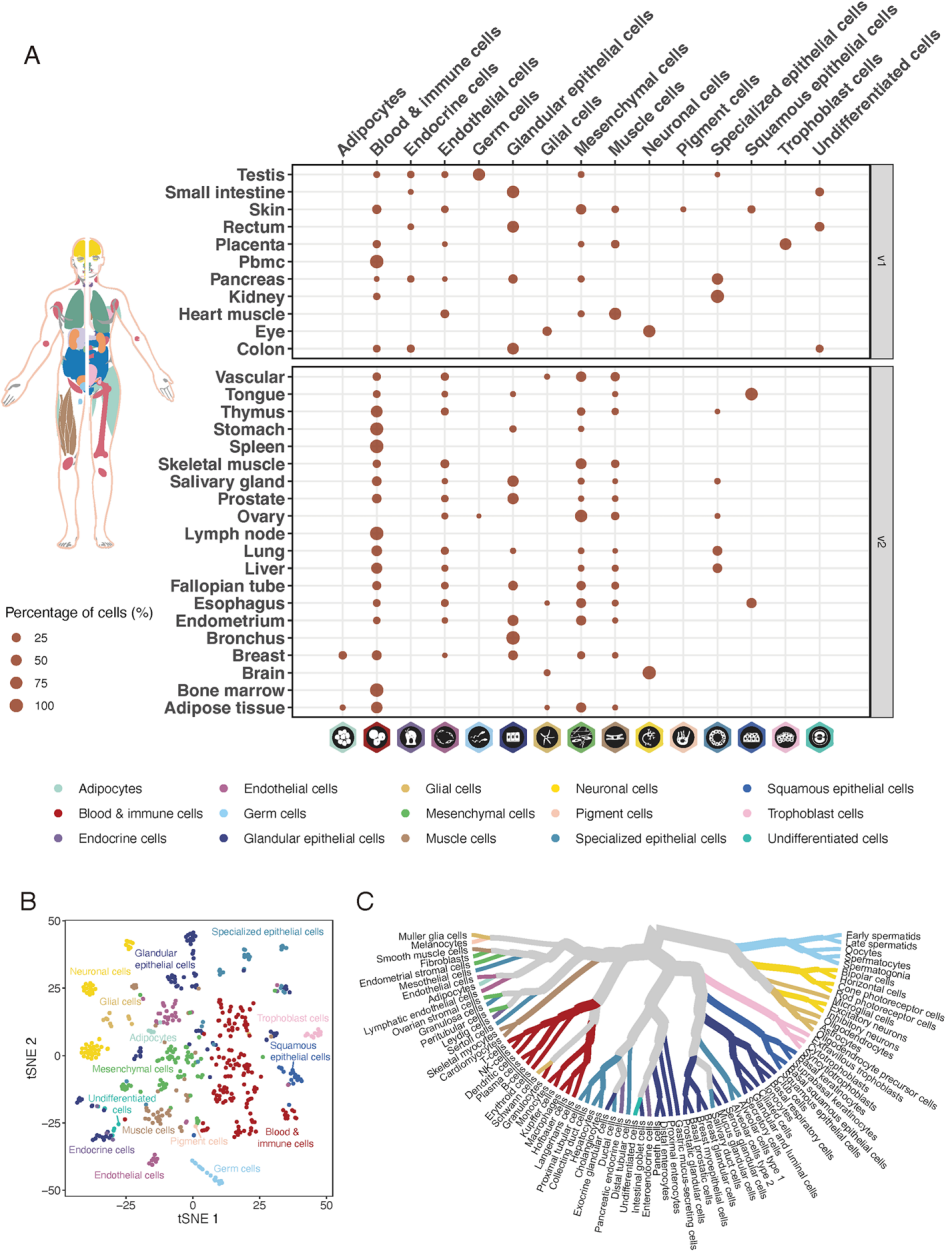
The transcriptional landscape of single cell types. **A** The bubble plot shows the percentage of cells of the 31 tissues in 15 cell type groups in this study. **B** tSNE plot shows the similarity of the pseudo-bulk transcriptomic expression profiles of the 518 cell type clusters. **C** Cluster tree plot shows the similarity of 81 cell types in terms of their transcriptomic expression profile where the distance between branches is decided by Spearman’s correlation. See also Figs. S1, S2, and S3 A in Additional File 2 and Table S2 in Additional File 1

In the updated version of the open access Single Cell Type section in the HPA database, single cell transcriptomics data from 31 distinct tissues (Table 1) are presented, which include datasets from 17 new organs and data sources, 3 existing organs with new data sources, and 11 existing organs with their original data sources. The newly added tissues include adipose [14], bone marrow [15], brain [16], breast [17], bronchus [18], endometrium [19], esophagus [15], fallopian tube [20], lymph node [15], ovary [21], salivary gland [2], skeletal muscle [22], spleen [15], stomach [15], thymus [2], tongue [2], vascular [2], and the tissues for which the datasets were replaced are liver [23], prostate [24], and lung [24] (Fig. 1A). This expanded tissue set encapsulates a variety of organ systems and crucial cell types, enabling a comprehensive single-cell analysis across a collective of 689,601 individual cells. This notably augments the gaps that remained unrepresented in the earlier version.

**Table 1:** Summary of dataset

Tissue	Data source	No. of M reads	No. of cells	No. of samples	References
Adipose tissue	GSE155960	351.1	80083	12	Hildreth AD et al. (2021) [14]
Bone marrow	GSE159929-GSM4850584	9.8	3484	1	He S et al. (2020) [15]
Brain	Allen brain	1403.9	76533	2	Allen brain map
Breast	GSE164898	262.1	46126	8	Bhat-Nakshatri P et al. (2021) [17]
Bronchus	fig11981034	87.9	26676	4	Lukassen S et al. (2020) [18]
Colon	GSE116222	47.1	5302	3	Parikh K et al. (2019) [25]
Endometrium	GSE111976	284.5	52594	10	Wang W et al. (2020) [19]
Esophagus	GSE159929-GSM4850580	33	10441	1	He S et al. (2020) [15]
Eye	GSE137537	12.6	9555	6	Menon M et al. (2019) [26]
Fallopian tube	GSE178101	416.4	62514	10	Ulrich ND et al. (2022) [22]
Heart muscle	GSE109816	55.8	6012	12	Wang L et al. (2020) [19]
Kidney	GSE131685	35.9	18365	2	Liao J et al. (2020) [27]
Liver	GSE115469	32.8	11175	5	MacParland SA et al. (2018) [23]
Lung	Tabula sapiens	349.6	27756	2	Tabula Sapiens Consortium* et al. (2022) [28]
Lymph node	GSE159929-GSM4850583	16.4	9076	1	He S et al. (2020) [15]
Ovary	E-MTAB-8381	144.4	37104	4	Wagner M et al. (2020) [21]
Pancreas	GSE131886	110	5313	3	Qadir MMF et al. (2020) [29]
PBMC	GSE112845	18.9	5274	1	Chen J et al. (2018) [30]
Placenta	E-MTAB-6701	326	25615	7	Vento-Tormo R et al. (2018) [31]
Prostate	Tabula sapiens	90.7	19009	2	Tabula Sapiens Consortium* et al. (2022) [28]
Rectum	GSE125970	44.2	2638	2	Wang Y et al. (2020) [19]
Salivary gland	Tabula sapiens	231.7	28809	2	Tabula Sapiens Consortium* et al. (2022) [28]
Skeletal muscle	GSE143704	61	24579	10	De Micheli AJ et al. (2020) [22]
Skin	GSE130973	57.4	22335	5	Solé-Boldo L et al. (2020) [32]
Small intestine	GSE125970	45.8	5460	2	Wang Y et al. (2020) [19]
Spleen	GSE159929-GSM4850589	15.6	4492	1	He S et al. (2020) [15]
Stomach	GSE159929-GSM4850590	20.4	5701	1	He S et al. (2020) [15]
Testis	GSE120508	65.2	6459	6	Guo J et al. (2018) [33]
Thymus	Tabula sapiens	197	23618	1	Tabula Sapiens Consortium* et al. (2022) [28]
Tongue	Tabula sapiens	283.7	18331	2	Tabula Sapiens Consortium* et al. (2022) [28]
Vascular	Tabula sapiens	172.5	9172	1	Tabula Sapiens Consortium* et al. (2022) [28]

The table summarizes the data sources for the 31 tissues. Detailed sample information, including accession numbers, sex, and age, is provided in Additional File 1: Table S1

To determine the quality of the dataset, we took into account several factors for each organ studied: the sequencing platform used, the total number of samples collected (Additional File 1: Table S1 for detailed information), the number of sequencing reads obtained, and the number of individual cells sequenced (see method and Table 1). Besides, we compared the pooled single cell transcriptomic profile with their corresponding bulk tissue profiles obtained from the HPA. A high Spearman’s correlation was observed between pseudo-bulk transcriptomic expressions and their corresponding bulk transcriptomic profiles, ranging between 0.75 and 0.90 (Additional File 2: Fig. S1 A). An exception was observed in the case of the brain with much lower correlation (*r* = 0.66).

Next, to obtain the cell type specific transcriptomic profiles, all single cell datasets have been mapped to the same reference genome for gene expression quantification (except for brain). For quality control at the single-cell level, we established specific thresholds for a range of parameters for each organ, such as number of detected genes, percentage of mitochondrial genes, and percentage of ribosome genes (Additional File 2: Fig. S1B–S1D, Additional File 1: Table S2). Cell type identification, annotation, and pseudo-bulk transcriptomic profiling of each cell type were executed according to a scheme delineated in Fig. S2 A (Additional File 2). Specifically, we implemented Louvain clustering to categorize similar expressing single cells within each tissue into distinct cell clusters. The number of cell clusters varied per tissue, ranging from 7 (lymph node) to 45 (brain). This process led to the body-wide identification of 557 unique cell clusters, which were manually annotated based on the expression of established cell type marker genes (Additional File 1: Table S3), and correlation with single-cell type protein expression patterns observed through immunohistochemistry. A reliability score (high, medium, low, very low) was assigned to each annotated cluster based on the marker genes, and clusters with very low and low reliability (*n* = 39) were excluded from subsequent analyses. Pooled data from each cluster were used to generate normalized transcripts per million (nTPM) for each gene. A detailed description of the data preprocessing steps is available in the supplementary methods (Additional File 3), including software resources, data selection, cell clustering, and cell type annotation [34–39].

By collating results from 31 tissues, we calculated the expression profile for each gene across the 518 single-cell type clusters. As many of the single-cell types, such as immune cells, neurons, fibroblasts, and endothelial cells, were identified across multiple tissues, these 518 single-cell type clusters were further aggregated into 81 consensus single-cell types. These were then classified into 15 different tissue groups based on shared functionalities within organ systems. We visualized all cells included (689,601 in total) using UMAP plots, which highlighted the clustering of cells with similar functions across the 31 organs and 15 cell type groups. This visualization, presented in the Fig. S2B and Fig. S2 C (Additional File 2), confirms that cells with analogous functions, such as immune cells, neurons, glial cells, and germ cells, tend to cluster together, regardless of their tissue origin. Additionally, cells from uniquely functioning organs also demonstrated close clustering, exemplifying the liver, brain, eye, and testis. In the open-access Single Cell Type section of the Human Protein Atlas (www.proteinatlas.org), the expression profiles (nTPM) for all genes are displayed across the individual 557 single-cell type clusters and the 81 consensus single cell types. This enables researchers to explore the single-cell profiles of protein-coding genes on a genome-wide scale.

We calculated the fraction of cells across each of the 31 tissues to investigate the overall distribution of the 15 primary tissue groups (Fig. 1A) and 81 cell types (Additional File 2: Fig. S3 A). The results revealed as expected that immune cells, particularly T-cells (*n* = 22), and smooth muscle cells (*n* = 18) were prevalent across most tissues. Conversely, 56 cell types, such as trophoblast cells, pigment cells, and several specialized cells, were identified as tissue specific. The overall similarity in cell type expression profiles was visualized via dimensional reduction analysis utilizing the t-distributed stochastic neighbor embedding (t-SNE) [40–43]. The t-SNE analysis (Fig. 1B) of all 518 reliable single-cell type clusters showed that clusters associated with unique tissue functions, such as intestinal, hepatic, brain, and placenta, had closely related profiles. Similarly, clusters from different tissues sharing similar functions, such as fibroblasts, smooth muscle cells, and immune cells, demonstrated high similarity in gene expression profiles. The similarity within the 81 consensus single-cell types was examined using Ward D2 [44] of hierarchical clustering method and visualized in a dendrogram [45–49] (Fig. 1C), resulting in 15 distinctive functional groups.

### Classification of protein-coding genes based on the extended single-cell type reveals novel cell type specific genes

**Fig. 2:**
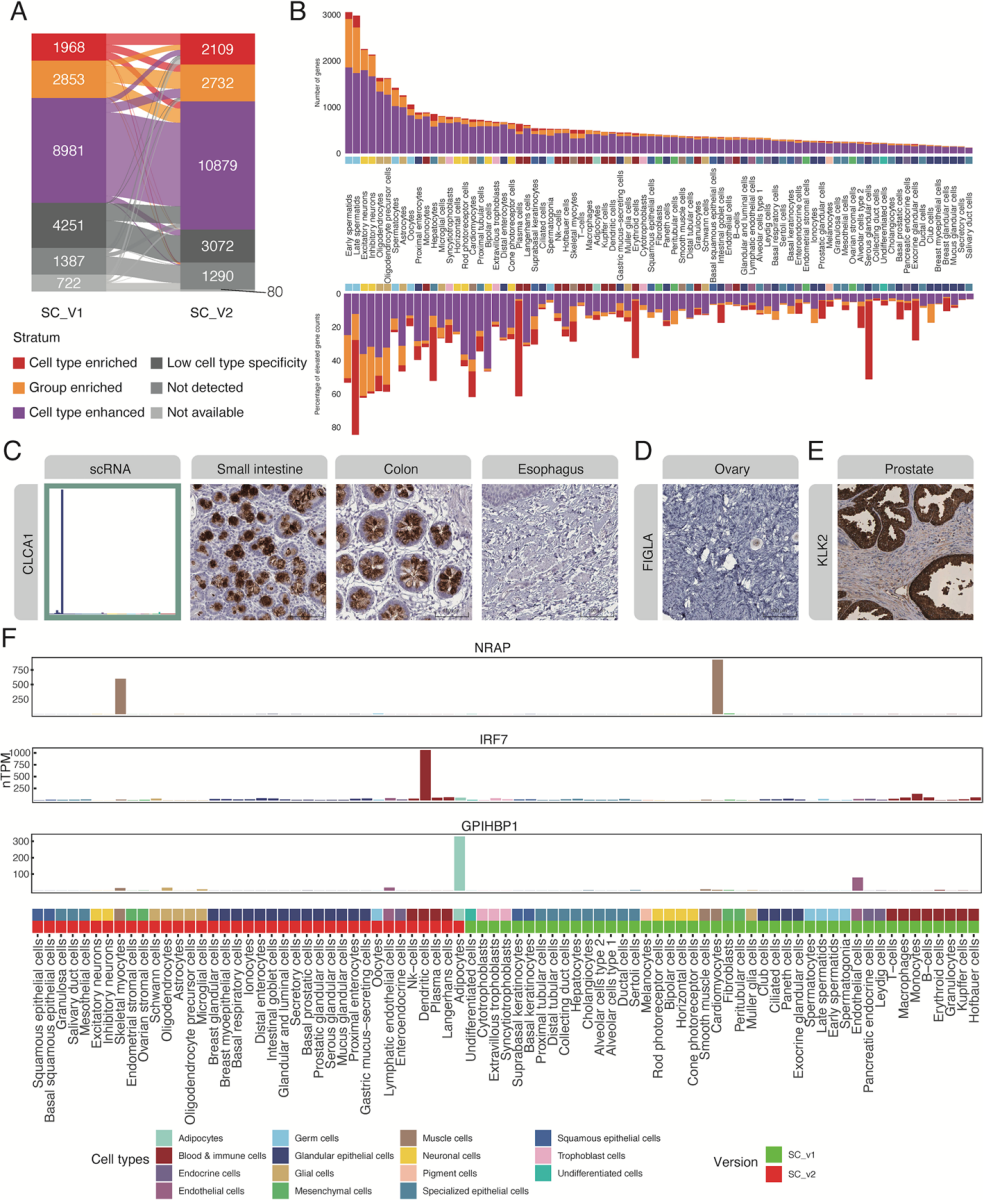
Classification of protein-coding genes based on the extended single-cell type reveals novel cell type specific genes. **A** The alluvial plot shows the number changes between gene classification defined in the current version and version 1 of the Single Cell Type Atlas. **B** The upper stacked bar plot shows the number of cell type elevated genes (enriched, grouped enriched and enhanced, respectively) of each cell type, while the lower stacked bar plot shows the proportion of these elevated gene counts for corresponding cell types. **C** The bar plot and IHC images show RNA expression of CLCA1 across 81 cell types and its protein staining in small intestine, colon, and esophagus, respectively. **D** The IHC image shows the protein staining of FIGLA in ovary. **E** The IHC image shows the protein staining of KLK2 in prostate. **F** The bar plots respectively show the RNA expression of NRAP, IRF7, and GPIHBP1 across 37 new cell types and 44 already existed cell types. Scale bar 100 µm. See also Fig. S4 in Additional File 1

To assess the specificity of gene expression across different cell types, we categorized all protein-coding genes based on their transcriptomic profiles. This classification allowed us to identify genes exhibiting various levels of specificity, including tissue-specific or cell-specific enrichment [5, 50, 51]. All 20,082 human protein-coding genes were evaluated concerning their mRNA expression profiles across the 81 identified single-cell types, following the criteria summarized in the supplementary methods (Additional File 3) [13, 35, 52]. Figure 2A [53] provides a detailed overview of the distribution of genes within each specificity category, as determined by the single-cell transcriptomic analysis previously conducted [5] and updated as part of this study.

In this classification, 2109 genes, representing 10% of the total number of genes, were identified as “cell type enriched,” indicating high specificity in a single cell type. A total of 2732 genes, or 14%, were classified as “cell type group enriched,” showing high enrichment within a group of up to 10 cell types. Furthermore, nearly half of the genes (*n* = 10,879) were categorized as “cell type enhanced,” displaying moderately elevated expression in at least one cell type. An additional 3072 genes exhibited “low cell type specificity,” comprising 15% of the analyzed genes. This important category of genes includes, among others, various “housekeeping” genes essential for fundamental cellular operations. Genes exhibiting expression below the detection limit (nTPM < 1) across all cell types were designated as “not detected,” corresponding to only 6% of all genes (*n* = 1290). It is important to note that the gene classifications presented here are based on available data, which may not cover all human cell types. For instance, genes primarily expressed in specialized tissues (such as olfactory genes) may be incorrectly classified as not expressed due to the absence of those tissues in our current database. Users should consider these limitations when interpreting the data, particularly for highly specialized cell types or tissues not represented in our collection. Hence, 18,712 genes were detected in at least one of the cell types analyzed in this study.

The body-wide, single cell type specificity of all genes was subsequently explored to classify each gene into categories, such as cell type enriched and group enriched. As depicted in Fig. 2B [42, 54, 55], testicular germ cells exhibit the highest count of enriched genes with 149 cell type enriched and 1049 group enriched genes, supporting earlier studies reporting many tissue enriched genes in testis [5, 50, 56, 57]. Late and early spermatids collectively display enriched expression in over 1000 genes, either specific to one or both cell types. This is likely attributable to their lack of transcriptional regulation during meiosis and the specialized function in the spermatogenesis process, a distinctive feature of male reproduction (Additional File 2: Fig. S4 A). Another example of a cell type exhibiting a significant number of elevated genes is the hepatocyte, with 148 cell type enriched genes, with an overrepresentation related to liver-specific functions, such as hemostasis, steroid metabolic processes, and lipid transport. Besides, only around 20% of the transcripts are encoded by cell type elevated genes, except for cell types responsible for exocrine functions, such as serous glandular cells, hepatocytes, and plasma cells (Fig. 2B).

Comparing the cell types in this study with those in the prior version, we have added 37 new cell types. These were identified using cell markers from both PanglaoDB [58] and our own classification, and these cell markers were also classified as elevated genes within their respective cell types and validated further through antibody-based profiling using human tissue samples. As an example, CLCA1, known as a marker gene for goblet cells and Paneth cells, was employed in our study as a marker gene for intestinal goblet cells (Fig. 2C). Similarly, FIGLA was employed as a marker for oocytes (Fig. 2D) and KLK2 for prostatic glandular cells (Fig. 2E), both validated by tissue staining.

The introduction of new cell types has prompted changes in gene specificity classification. A comparison (Fig. 2A) between the specificity classification of cell types in this study and the previous version reveals a decrease in the number of “low specificity,” “not detected,” and “not available” genes. However, there is an increase in the category of “enhanced” genes, which likely reflects the larger pool of genes sourced from the expanded dataset and inclusion of more cell types. Moreover, over half of the “cell type enriched” genes identified in the previous study have become less specific by the introduction of more tissues in the current study. For example, NRAP, initially classified as a cardiomyocyte enriched gene, is now categorized as a group enriched gene due to the recent addition of skeletal myocytes as a cell type (Fig. 2F). This shift suggests that as we include more cell types, we find that genes once considered unique to one cell type are also expressed in others with biological meaning. Conversely, we also discovered that as many as half of the newly classified cell type enriched genes were previously defined as group enriched, cell type enhanced, or had no cell type specificity. For instance, IRF7 and GPIHBP1, which now show exceedingly high expression in the newly included cell types dendritic cells and adipocytes, respectively (Fig. 2F).

Overall, the current version of Single Cell Type section contains more cell type elevated genes, underscoring that the expanded analysis of single-cell type datasets offers a more comprehensive transcriptomic landscape of diverse cell types. This enhanced resolution is anticipated to provide a more accurate depiction of the body-wide, gene-centric cell type specificity in humans.

### Comparison of bulk and single cell transcriptomics

To augment the specificity classification detailed earlier, we chose to use the tau specificity score [24], a measure previously proven to score gene expression specificity both accurately and robustly [59]. Tau assigns each gene a score from 0 to 1 based on its expression specificity: a tau of 0 indicates uniform expression across all cells or tissues, while a tau of 1 signifies expression exclusive to a single cell or tissue [24]. Examining the single cell transcriptomics dataset, we observed tau scores ranging from 0.1 to 1.0, showing a high degree of concordance with our specificity categorization scheme (polyserial correlation [60]: 0.82, Additional File 2: Fig. S4B, Additional File 3: supplementary methods). Genes classified by us as enriched in a specific cell type exhibited a median tau score of 0.95, whereas group enriched genes scored 0.90, cell type enhanced genes 0.58, and genes with low cell type specificity 0.28. The bulk transcriptome dataset showed a relatively similar trend (polyserial correlation: 0.85, Additional File 2: Fig. S4B), where the median tau score of tissue enriched genes, group enriched genes, tissue enhanced genes, and low tissue specificity genes is 0.93, 0.83, 0.60, and 0.28, respectively. It should be noted that, although genes generally exhibit similar specificity trends in both datasets, with a Spearman correlation of tau equal to 0.90 (Additional File 2: Fig. S4 C), the tau score in single cell data is significantly higher than that in bulk tissue data (Additional File 2: Fig. S4D, *P* value < 1e − 6).

**Fig. 3:**
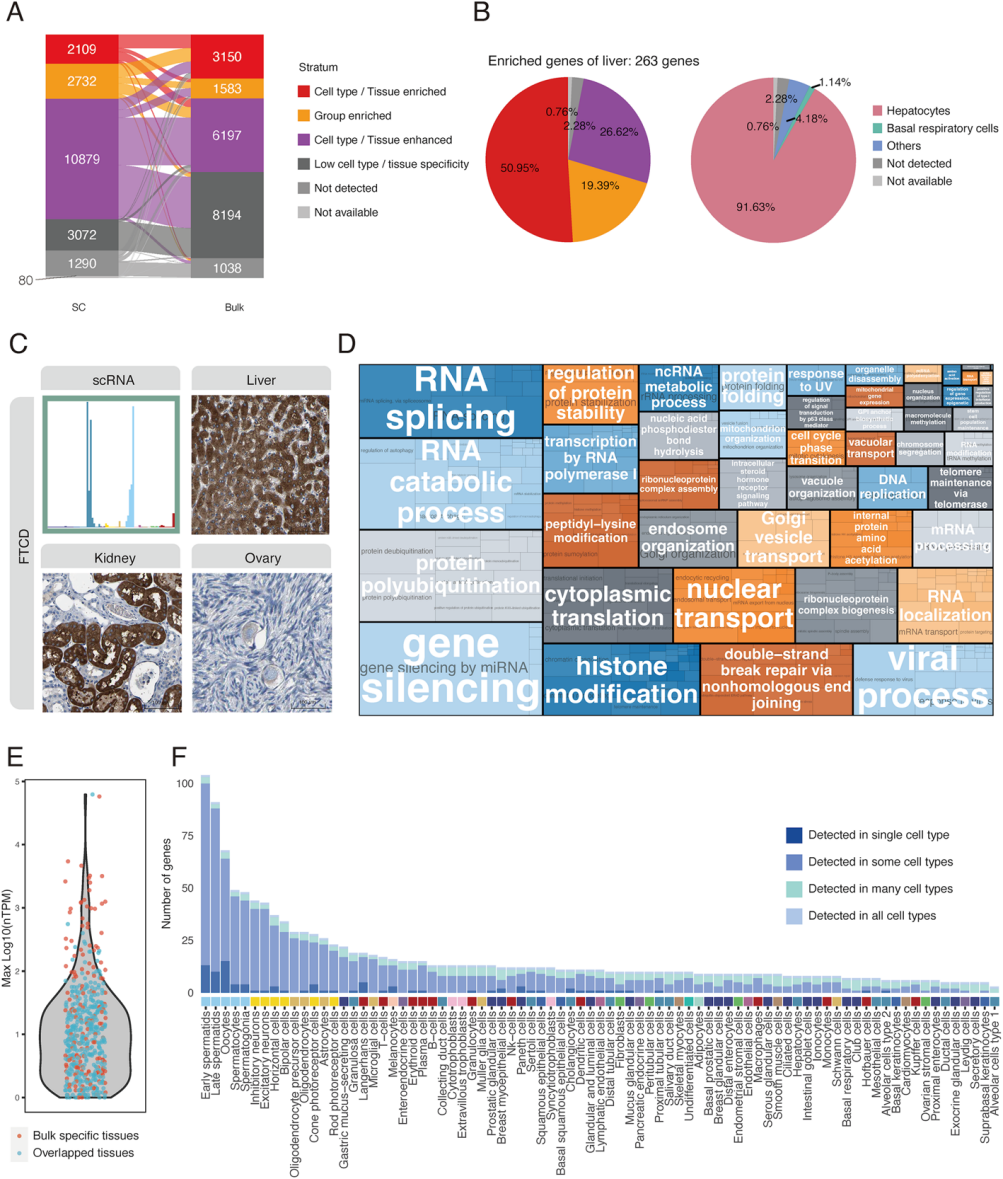
Comparison of bulk and single cell transcriptomics. **A** The alluvial plot shows the number changes between gene classification based on single cell and bulk transcriptomics. **B** The pie chart shows the gene classification of all the liver enriched genes based on single cell transcriptomics, and the elevated cell types of all the liver enriched genes. **C** The bar plot and IHC images show RNA expression of FTCD across 81 cell types and its protein staining in hepatocytes (liver), proximal tubular cells (kidney) and oocytes (ovary), respectively. **D** The tree map shows the enriched parent GO BP terms of genes with consensus low specificity in both gene classifications based on single cell type and tissue transcriptomics. **E** The violin plot shows the maximum RNA expression of genes that are only detected in bulk transcriptomics of tissue samples in HPA. The red and blue colors respectively indicate that if the tissue that has the maximum expression level of the genes is bulk specific tissues or not. **F** The bar plot shows the distribution of genes across 81 cell types that are only detected in the single cell transcriptomics dataset. Scale bar 100 µm. See also Fig. S4 in Additional File 1

In order to examine the shift in gene specificity classification between single cell and corresponding bulk transcriptomics datasets, we conducted a comparison for all genes. As shown in Fig. 3A, single cell transcriptome data hosts a larger number of elevated genes, corresponding to the higher tau scores mentioned earlier. However, genes that were “tissue enriched” showed less specificity in single cell transcriptomics, with more than two-thirds being classified as either group enriched or cell type enhanced genes. This outcome is expected and can be attributed to the fact that bulk data comprises several cell types identical in different tissues and the bulk data is thus influenced by the composition of these cell types. This is exemplified by liver enriched genes (*n* = 263), wherein only half are categorized as cell type enriched genes (Fig. 3B). Interestingly, over 90% of these genes are found to be elevated in hepatocytes (Fig. 3B), thus exemplifying genes that are enriched in multiple cell types, but confined to a single tissue, likely due to the fact that hepatocyte comprises approximately 80% of total liver tissue [61]. Additionally, an illustration of genes enriched in multiple cell types but confined to a single tissue is provided by FTCD (Fig. 3C). This gene is group enriched, showing similar expression levels in hepatocytes, proximal tubular cells, and oocytes. However, due to the varying population of these cell types, only the liver displays enrichment of FTCD.

Another category showing significant variation in composition is the low specificity genes (genes expressed in all). Among the 8194 genes showing low tissue specificity, over 60% are classified as cell type enhanced genes in the single cell transcriptome. The enriched functions of the 2826 genes consistently exhibiting low specificity were analyzed via Gene Set Overrepresentation Analysis (GSOA) and displayed in a tree map (Fig. 3D) [62, 63]. These primarily encompass basic cellular functions like transcriptome and proteome modification. In contrast, the enriched functions of the remaining 5368 genes tend to focus on processes requiring cell–cell interaction, such as signaling pathways and cell migration (Additional File 2: Fig. S4E). This delineates the variation in “housekeeping” genes at the level of cell types and tissues, and provides us with a list of genes that play roles in fundamental cellular functions.

In addition to this, single cell transcriptomics data displays a greater number of undetected genes (*n* = 1290), with 586 of these genes detectable in HPA bulk data. The main reason for this discrepancy is the larger data size of the bulk transcriptomics data in HPA, encompassing not only more sample subjects, but also a wider variety of tissues. The maximum expression levels of these genes among tissues in bulk data is shown in the violin plot (Fig. 3E), where over 90% of the genes exhibit nTPM values less than 100. Conversely, 307 genes are detected exclusively in single cell transcriptomics. These genes are of particular interest as they represent expression patterns not reflected in bulk tissue data, thereby underscoring the added information afforded by single cell-based analysis. When mapping these genes to our single cell transcriptomics data, we found that 93 genes are detected only in a single cell type; 208 genes are detectable in some cell types; 5 genes are detectable in many cell types; and 1 gene is detected in all cell types (Additional File 2: Fig. S4 F). Overall, these genes are most frequently detected in single cell types present in single tissues with relatively low abundances (Fig. 3F). Thus, the specific expression in single cell types can often be obscured in bulk transcriptomics analysis, as exemplified by genes expressed in spermatids in the testis and oocytes in the ovary.

The shift in gene specificity classification between bulk and single-cell transcriptomics data emphasizes the nuanced insights provided by single-cell analysis. Single-cell data excels in detecting unique genes, especially those expressed in rare cell types, thus uncovering previously unrecognized expression patterns. In contrast, bulk data provides broader gene coverage, particularly for low-abundance genes, underscoring the complementary nature of both approaches in capturing the full spectrum of gene expression.

**Fig. 4:**
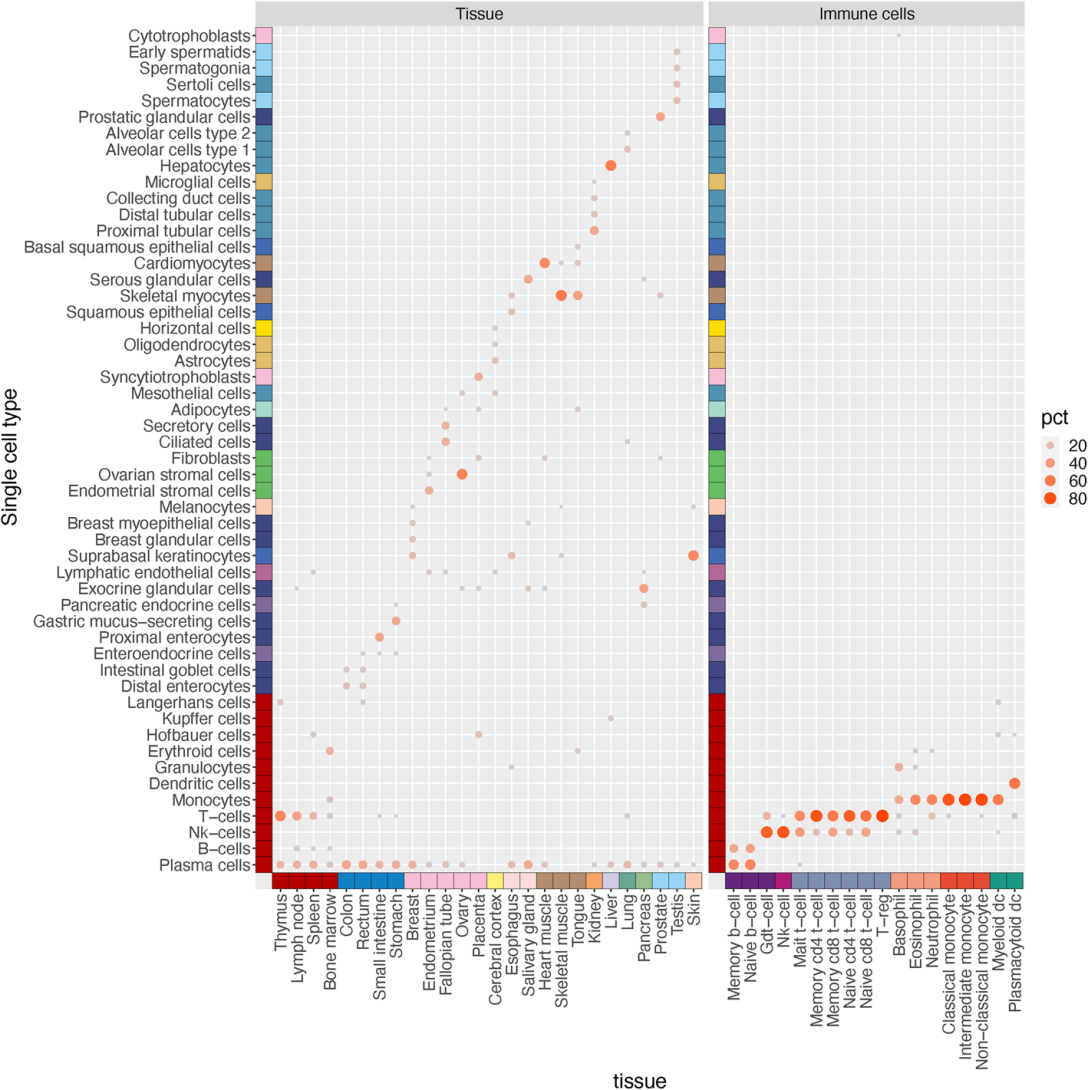
Deconvolution of bulk transcriptomic data. The bubble heatmap shows the predicted cell composition of 25 bulk tissues and 18 immune cell transcriptomics. For each tissue and organ, the top 5 cell types are shown. See also Fig. S3B in Additional File 2

Next, we performed deconvolution of bulk transcriptomics from the tissue atlas. Utilizing pseudo-bulk data from 81 cell types as single-cell reference data and 2109 cell-type enriched genes as signature genes, the cell type composition of bulk data was estimated by employing dampened weighted least squares (DWLS) [64]. Detailed information is available in supplementary methods (Additional File 3). Figure 4 demonstrates the top 5 cell-type compositions for each tissue as well as immune cell. As expected, the bulk data comprised specific single cell types, such as T-cells in the thymus, proximal enterocytes in the small intestine, skeletal myocytes in skeletal muscle, cardiomyocytes in heart muscle, and hepatocytes in the liver. This observation indicates that the cell-type enriched genes successfully captured the characteristics of the cell types.

Additionally, we conducted hypergeometric tests between enriched genes in all cell types and genes that enriched in tissues and immune cells. The results are displayed in a bubble heatmap (Additional File 2: Fig. S3B), where each bubble corresponds to a statistically significant enrichment. It is encouraging to note that single cell types specific to a particular tissue—for instance, cardiomyocytes (heart), germ cells (testis), ciliated cells (fallopian tube), and trophoblasts (placenta)—show high enrichment in their corresponding bulk tissues when analyzed with single cell transcriptomics. This suggests that these genes consistently display high specificity. Cells with analogous functions overlap with similar tissues. For example, both keratinocytes and squamous epithelia of the skin overlap with tissues such as urinary bladder, vagina, esophagus, and skin. Hepatocytes primarily overlap with liver tissue, but some overlap with kidney tissue is also noted due to the shared group-enriched genes between the two.

Cardiomyocytes show overlap with bulk transcriptomics analysis of heart muscle, skeletal muscle, and tongue—all of which contain striated muscle cells—while myocytes overlap with skeletal muscle and tongue tissue. This demonstrates that despite many similarities among different smooth muscle cells, skeletal myocytes and cardiomyocytes maintain unique expression profiles. Furthermore, various glandular cell types overlap both with their tissue of origin and other glandular tissues. For instance, enteroendocrine cells overlap with gastrointestinal tissues and other organs with endocrine functions such as the pancreas and pituitary gland. As for immune cells, as expected, T-cells, myeloid cells, and B cells show clear partition to each other, supporting their high heterogeneities.

## Utility and discussion

### User interface overview

The Human Protein Atlas v24 (https://v24.proteinatlas.org), as an open access knowledge resource, covers human protein profiles in cells, tissues, organs, and blood. Single cell type, as one of the main resources of the HPA, offers a user-friendly and intuitive interface with more than 1,000,000 interactive plots to allow researchers to explore the expression in individual single cell types for all protein-coding genes in these tissues. Key design elements include:

i) Introduction page (https://v24.proteinatlas.org/humanproteome/single+cell/single+cell+type): A knowledge summary to show the proteomes of the single cell types with four main sections of information. The first box briefly describes the study design of the Single cell type resource, providing links to detailed information such as description of datasets, methodologies, and comparison with another database (Tabula Sapiens), and two clickable genes (CNN1 and TSPY2) with thumbnail visual examples of the information found in the resource. The second box contains the proteomes of the single cell types in pages. Each page is dedicated to a cell type group and each group includes cell types with functional features in common. Symbols and names are clickable and will redirect to the corresponding page. If users hover the mouse pointer over a certain symbol or name, the corresponding location in the human body will be highlighted in the human figures. Additionally, a tree-like cell type dendrogram is designed to show the relationship between all single cell types based on genome-wide expression. Each cell type is clickable and will redirect to the corresponding section of the cell type proteomes. The dendrogram is based on agglomerative clustering of 1– Spearman’s rho between cell types using Ward’s criterion, and then transformed into a hierarchical graph, thus where link distances were normalized to emphasize graph connections rather than link distances. Link width is proportional to the distance from the root, and are colored according to cell type group if only one cell type group is present among connected leaves. The third box is a UMAP that is generated by clustering genes based on expression patterns. The colored areas represent the area in the UMAP where most of the genes of each cluster reside. Clicking on a cluster will redirect to view an interactive UMAP and details about all cluster annotations. The last box provides a more detailed explanation of gene classification in single cell transcriptome in three formats: pie chart, bar plot, and table. Users can see the distribution of genes with elevated expression across various cell types in this table. All elements are clickable and redirect to associated lists of genes or proteome knowledge summary page.
ii) Search bar: Positioned at the top, allowing users to quickly search for specific genes or proteins across the entire dataset. Advanced search service is provided to limit the field of search.
iii) Overview dashboard: In the summary page of a specific gene, a dashboard of cell type RNA expression is provided with a clean, visual summary showcasing key information, including gene classification, expression cluster, and aggregated gene expression bar plot. The bar plot is clickable and will redirect to the Single Cell resource with full details.
iv) Gene pages: Upon selecting the Single Cell resource of a specific gene, users are directed to a detailed page containing results of different assays including Single cell type, Tissue cell type enrichment, Single nuclei (brain), Immune cells and Expression clustering. Focusing on the Single cell type, a summary of gene nTPM values from all single cell types is presented through a combined visualization of bar and bubble plots. The title of this combined plot, placed at the top, highlights the gene’s specificity. The bar plot then displays the mean nTPM values of the gene across various cell types, providing a clear comparison. Below, the bubble plot complements this by illustrating the nTPM values in different tissues, with the size of the bubbles indicating expression intensity. The exact nTPM values will be showed in a floating box if users hover the mouse-pointer over a certain bar or bubble. Clicking a tissue name in the bubble plot or selecting it in the sidebar of the page will redirect to the tissue page in single cell type resource.
v) Tissue pages: Upon selecting a specific gene and a specific tissue in Single cell type, users are directed to a detailed page of the tissue. Basic information includes two parts. The first one is the expression profile of the gene in the tissue, visualized by a UMAP plot and a bar chart. The UMAP plot visualizes the cells in each cluster; where each dot corresponds to a cell. For each individual cell, mouseover reveals read count and which cluster the cell belongs to. Hovering over a cluster name will highlight the corresponding cluster in the bar chart below. There are 2 options for color schemes: (1) cell type color, which is based on cell type groups used in the single cell type part of the single cell resource and (2) cluster color, which assigns a unique color to each cluster. The cells are colored according to % of max (log2(read_count + 1)/log2(max(read_count) + 1)*100) in five different bins (< 1%, < 25%, < 50%, < 75%, ≥ 75%). There is also a read count filter to hide cells below a certain read count in the UMAP. The bar chart, combining with the legend of the UMAP plot, shows RNA expression (nTPM) in each cell type cluster. Hovering over the cluster name reveals nTPM value and number of included cells. Hovering over a bar highlights the corresponding cluster in the UMAP plot above. Color-coding can be toggled on the top of the page, between 2 options: (1) cell type color, which is based on cell type groups used in the single cell type part of the single cell resource and (2) cluster color, which assigns a unique color to each cluster. For the 19 tissues that overlap with Tabula Sapiens (lung, prostate, salivary gland, thymus, tongue, vascular, adipose tissue, bone marrow, eye, heart muscle, kidney, liver, lymph node, pancreas, skeletal muscle, skin, small intestine, spleen), we serve a similar design but with expression data and cell type annotation from Tabula Sapiens [2]. The second part of the page is a heatmap showing expression of the currently selected gene (on top) and well-known cell type markers in the different single cell type clusters of this tissue. The panel on the left shows which cell type each marker is associated with. Hover the mouse pointer over the individual data points (squares) to see nTPM level and *Z*-score. Clicking on a gene name redirects to the corresponding gene page. *Z*-score is when you normalize a variable such that the standard deviation is 1 and the mean is 0. Thus, all the genes are easier to compare, as they have the same center and distribution. It is easy to move between the different tissue detail pages by simply clicking the tissue name and three dashes in the left-hand panel and picking a different tissue type; here, it is also visualized with color dots if Tabula Sapiens data is shown.
vi) Data description and downloading: Clicking the “data” link on the home page of Single cell type, users will be redirected to the page where the single cell type data is described and downloaded in three layers. The first layer is based on tissue dataset, illustrating the tissue name, data source, total cell numbers, read counts and reference link. The second layer is based on cell type data, illustrating the 81 cell types and their corresponding cell type group. The third and last layer is based on cluster data, illustrating the 557 clusters and their corresponding tissues, cell types, cell type groups, cell counts, if included in aggregation and the reliability of annotation.
vii) Methodology: Clicking the “Method summary” link in the home page of Single cell type, users will be redirected to the page describing the method details of data analysis. The page answers key questions such as key publications, what can users learn, how has the data been generated and analyzed, what is presented, etc. The page features an easy-to-understand workflow, complemented by clear equations used in the calculations. This combination ensures that users can follow the logical steps of the process while having access to the underlying mathematical principles, enhancing both clarity and comprehension. Additionally, users can easily navigate to topics of interest by clicking on the index located in the sidebar.
viii) Cluster comparison: Clicking the “A cluster comparison between the HPA pipeline and Tabula Sapiens can be found here” link in the home page of Single cell type, users will be redirected to the page providing cluster and dataset comparison between HPA Single cell type and Tabula Sapiens. The page includes several main sections such as inclusion criteria for data in single cell type resource, brief description of Tabula Sapiens, overlapped tissues in our resource and Tabula Sapiens, and one example gene for each tissue.
ix) Cell type specific proteome: Clicking a cell type group in the home page of Single cell type, users will be redirected to the corresponding page of the cell type group, which contains detailed information about the cell type specific proteome. Generally, the page starts with an overview of the cell type group describing its functions, location, number of enriched or elevated genes, and sub-cell types. For each of the sub-cell types, at least one of the cell type enriched genes will be exemplified by showing its mRNA expression level in single cell type via UMAP plot and bar plot, and the protein expression level in single cell type via immunohistochemically stained tissue sections. This visualization can provide users the cell morphology of the cell type. References are listed at the end. Tissues that contain the cell type group are listed in the side bar.
x) Others: Several functions related to single-cell data are integrated within the HPA platform, such as data downloading, explanations of special terms, reports of release history, news, publications, and helping desk.

Notably, unless otherwise specified, the color-coding in single-cell type visualizations is consistently based on cell type groups. Each group is composed of cell types that share common functional characteristics, ensuring a logical and structured representation of the data.

### Intended uses of the database and envisioned benefits

HPA Single Cell Type is designed to complement the understanding of protein-coding gene expression across various cell types in the human body, with a broad range of applications for both academic researchers and clinicians. Gene expression exploration is a fundamental application of the platform, where its layered design allows users to systematically examine a gene’s expression at multiple levels: from broad cell type expression across the body, to organ-specific cell types, and finally to individual cells. The gene summary pages provide a general overview at first glance, while facilitating easy navigation into other areas for more detailed information. Users can easily compare gene expression patterns across cells, clusters, tissue-specific cell types, and body-wide cell types. The resource includes several knowledge summary pages to help users explore the data; the overlap between genes classified as tissue-specific versus cell type-specific is, for example, described (https://v24.proteinatlas.org/humanproteome/tissue/tissue+specific).

The platform integrates antibody-based protein validation, offering an essential layer of verification for gene expression, which is particularly valuable for translational research and experimental validation. Additionally, HPA Single Cell Type serves as an educational resource, offering detailed information on cell types and groups through both text and imagery. All data are available for free download without registration, making it a valuable data repository for advanced research, including applications in machine learning and AI model development.

### Benchmarking with existing databases

The HPA Single Cell Type section offers several distinct advantages over existing single-cell transcriptomic databases. Notably, it aggregates single-cell transcriptomic data into pseudo-bulk across a comprehensive range of human tissues and organs, providing a more complete and robust dataset for understanding whole body protein-coding gene expression patterns. Compared to databases like the Human Cell Atlas [1], Tabula Sapiens [2], Single Cell Atlas [65], GTEx Portal [66], PanglaoDB [58], Single Cell Portal [67], Ensembl Single Cell Expression Atlas [68], and DISCO [69], the HPA Single Cell Type section extensively and uniquely analyzes key features of gene expression profiles across the whole body (Additional File 1: Table S4). These include gene specificity classification, categorizing genes by expression in specific cell types; Tau scores, which quantify gene specificity as continuous variables; gene detectability, providing insights into expression presence across various cell types; and gene clustering analysis, identifying groups of genes with similar expression patterns. These features offer a comprehensive view of protein-coding genes across the human body at the single-cell level, allowing systematic gene exploration.

Additionally, HPA emphasizes protein-level validation through antibody-based profiling, offering visual confirmation of gene expression within intact tissue samples and revealing cell morphology of the corresponding cell types. For the 19 tissues overlapping with Tabula Sapiens, the data from both platforms are shown in parallel, serving as additional references. HPA also excels in user experience, offering highly interactive features like customizable UMAP plots and strategic hyperlinks that facilitate the detailed exploration of gene expression and cell type descriptions. This comprehensive and intuitive design positions the HPA Single Cell Type section as a leader in single-cell transcriptomic databases.

### Future development and maintenance

To ensure the platform remains its continued relevance, usability, and expansion, the update of the HPA Single Cell Type section will be implemented annually. This commitment will enhance the platform’s utility and ensure it reflects the latest advancements in the field. Continuous incorporation of new scRNA-seq datasets from replicated as well as additional tissues and organs will be a priority. This will enhance the comprehensiveness of the atlas and reflect the most up-to-date research findings, but the batch effects could be a challenge. Moreover, future updates will aim to integrate multi-omics data types at single cell level, such as spatial transcriptomics, proteomics (SCoPE, CyTOF, CITE-seq, etc.), epigenomics (scATAC, etc.). This integration will enable users to analyze gene expression in conjunction with protein levels, epigenetic modifications, and spatial context within tissues, providing a more comprehensive understanding of cellular functions and interactions. Besides, aiming at improving user experience, we will enhance the UI design accordingly. All updates will be unified with the HPA platform, such as included protein coding genes, ensembl version, categories of gene specificity, color-coding and so on.

## Discussion

One distinguishing aspect of the body-wide single cell type map presented here, as compared to other studies, is its focus on pseudo-bulk transcriptomic gene expression across different cell types. By pooling cells with similar transcriptomic profiles, we aim to achieve a robust and comprehensive transcriptome for each well-defined cell type, albeit at the expense of losing single-cell level information. 81 cell types have been identified here, but this might be expanded when more knowledge on markers for different cellular subsets is obtained, or datasets corresponding to specialized tissues or tissues for which the gene expression varies due to temporal changes (e.g. fetal development or the menstrual cycle) have become available. Therefore, this approach could be improved once more specific markers for sub-cell type populations are identified [70, 71].

The brain is among the most complex tissue types harbouring many cell types with different population sizes and transcriptional activity. Using unbiased snRNAseq approaches requires enormous amounts of isolated nuclei to obtain a large enough sample of all cell types with enough statistical power to define the molecular signature of all cell types. This incompleteness drives the poor correlation between bulk and single nuclei data in the brain. We decided to include the data not only because it provides unique cell types, but also gives valuable and complementary information about the expression profiles of the human brain and the relationship of these profiles to the other organs and tissues in the human body, which are important for a representative single cell type map of the whole human body.

In summary, we describe the efforts to create an open access resource suitable for creation of AI-based cell models, in which transcriptome data using data from external and internal efforts have been integrated with data obtained using both single cell analysis and bulk tissue transcriptomics (RNAseq). 557 unique cell types have been identified and all genes have been classified into categories based on body-wide expression to provide visual confirmation of single cells within intact tissue samples. All the results have been added to the updated open-access Single Cell Type section of the Human Protein Atlas (www.proteinatlas.org) to facilitate genome-wide exploration of individual genes for further studies using AI-based machine learning with the long-term objective to understand and model the human cells in various tissues and organs.

## Conclusion

In this study, 17 new tissues and 37 new cell types have been added to the new Single Cell Type section of the Human Protein Atlas thereby broadening our knowledge of cellular diversity and the intricate transcriptomic landscape. This extensive coverage allows for a more nuanced and detailed mapping of cell type-enriched and housekeeping genes across a wide spectrum of human tissues. This breadth of analysis underpins the novel insights into cellular functions and gene regulation, offering an open access resource for future biomedical research and therapeutic interventions. Overall, our results emphasize the utility of single-cell transcriptomics in revealing a more detailed, gene-centric perspective of cell type specificity. All data is freely accessible in a separate section of the updated version of the HPA database (www.proteinatlas.org/celltype), thereby making it a publicly available tool for exploring individual single-cell type data for all protein-coding genes in these tissues and cell types.

## Supplementary Information

Additional File 1: Table S1. Summary of dataset sample information. Related to method sectionand Table 1. Table S2. Summary of parameters using in single cell processing. Related to method sectionand Figure S2. Table S3. Cell type markers. Related to Figure 2. Table S4. Comparison of HPA single cell type and other existing singel cell transcriptomic databases

Additional File 2: Fig S1. Quality control for single cell transcriptomics data, related to Figure 1.The bubble heatmap shows the spearman correlation between matched pseudo bulk and bulk transcriptomics of 25 tissues. Violin plots show the quality of cells for each tissue and organ in aspect ofnumber of genes by counts,mitochondrial gene percentageestimated doublet scores. Fig S2. Overview of the study, related to Figure 1.The workflow of HPA single cell section. Whole cell UMAP plot visualization on cell type groupsand tissues. Fig S3. Overview of the 81 cell types, related to Figure 1 and Figure 4.The bubble plot shows the percentage of cells of 31 tissues in 81 cell types.The bubble heatmap shows the enrichment of enriched genes of cell types in bulk and immune cell transcriptomics. Fig S4. Gene classifications, related to Figure 2 and Figure 3.The bubble heatmap shows the enriched GO BP terms of spermatid enriched genes.The violin plot shows the gene classification of genes and their corresponding tau score in single cell transcriptomics. The scatter plotand the box plotshows the similarity and differences of tau scores of consensus genes between single cell and bulk transcriptomics, respectively.The tree map shows the enriched parent GO BP terms of bulk-only low specificity genes.The bar plot shows the distribution of genes that only detected in single cell transcriptomics

Additional File 3: Supplementary methods

## Data Availability

The data supporting the findings of this study are available in our open-access HPA database (https://www.proteinatlas.org/about/download) and the supplemental information of the manuscript. The code is publicly available on github at https://github.com/MengnanShi/HPA_SingleCellSection.git under the MIT License [72]. It is also archived on Zenodo at https://zenodo.org/records/14007779 [73].
