# Supplementary material for: A resource for whole-body gene expression map of human tissues based on integration of single cell and bulk transcriptomics
Source: Genome Biol. 2025 Jun 3;26:152. doi: 10.1186/s13059-025-03616-4 (PMC12131445; doi:10.1186/s13059-025-03616-4)
Supplement: Supplementary file 2 — Additional File 2: Fig S1. Quality control for single cell transcriptomics data, related to Figure 1.The bubble heatmap shows the spearman correlation between matched pseudo bulk and bulk transcriptomics of 25 tissues. Violin plots show the quality of cells for each tissue and organ in aspect ofnumber of genes by counts,mitochondrial gene percentageestimated doublet scores. Fig S2. Overview of the study, related to Figure 1.The workflow of HPA single cell section. Whole cell UMAP plot visualization on cell type groupsand tissues. Fig S3. Overview of the 81 cell types, related to Figure 1 and Figure 4.The bubble plot shows the percentage of cells of 31 tissues in 81 cell types.The bubble heatmap shows the enrichment of enriched genes of cell types in bulk and immune cell transcriptomics. Fig S4. Gene classifications, related to Figure 2 and Figure 3.The bubble heatmap shows the enriched GO BP terms of spermatid enriched genes.The violin plot shows the gene classification of genes and their corresponding tau score in single cell transcriptomics. The scatter plotand the box plotshows the similarity and differences of tau scores of consensus genes between single cell and bulk transcriptomics, respectively.The tree map shows the enriched parent GO BP terms of bulk-only low specificity genes.The bar plot shows the distribution of genes that only detected in single cell transcriptomics [file 13059_2025_3616_MOESM2_ESM.pdf]

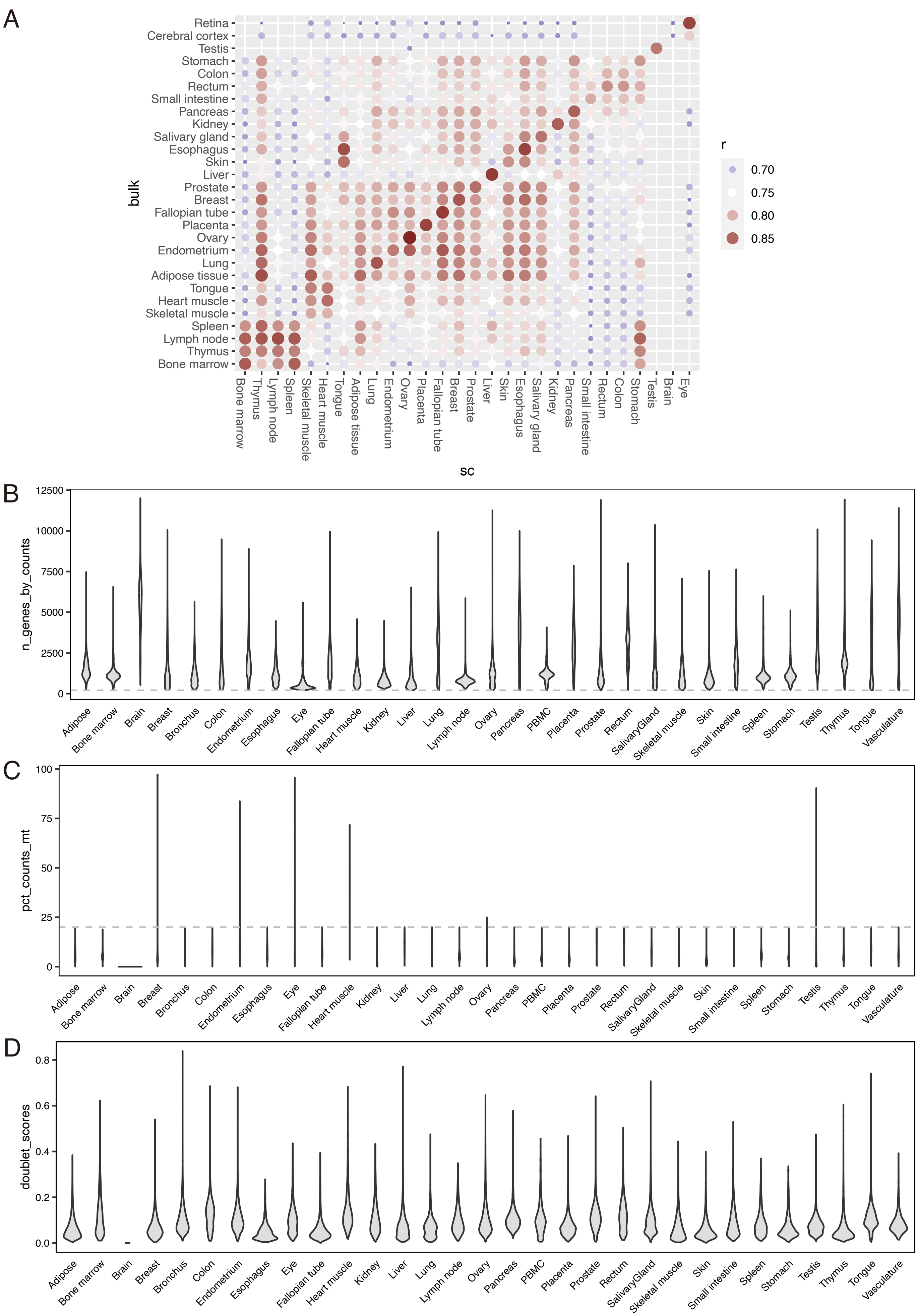
A
B
C
D
bulk
sc
r
0.70
0.75
0.80
0.85
Retina
Cerebral cortex
Testis
Stomach
Colon
Rectum
Small intestine
Pancreas
Kidney
Salivary gland
Esophagus
Skin
Liver
Prostate
Breast
Fallopian tube
Placenta
Ovary
Endometrium
Lung
Adipose tissue
Tongue
Heart muscle
Skeletal muscle
Spleen
Lymph node
Thymus
Bone marrow
Brain
Eye
n_genes_by_counts
pct_counts_mt
doublet_scores
Adipose
Bone marrow
Brain
Breast
Bronchus
Colon
Endometrium
Esophagus
Eye
Fallopian tube
Heart muscle
Kidney
Liver
Lung
Lymph node
Ovary
Pancreas
PBMC
Placenta
Prostate
Rectum
SalivaryGland
Skeletal muscle
Skin
Small intestine
Spleen
Stomach
Testis
Thymus
Tongue
Vasculature

Fig S1. Quality control for single cell transcriptomics data, related to Figure 1.

(A)The bubble heatmap shows the spearman correlation between matched pseudo bulk and bulk transcriptomics of 25 tissues. Violin plots show the quality of cells for each tissue and organ in aspect of (B) number of genes by counts, (C) mitochondrial gene percentage (D) estimated doublet scores.

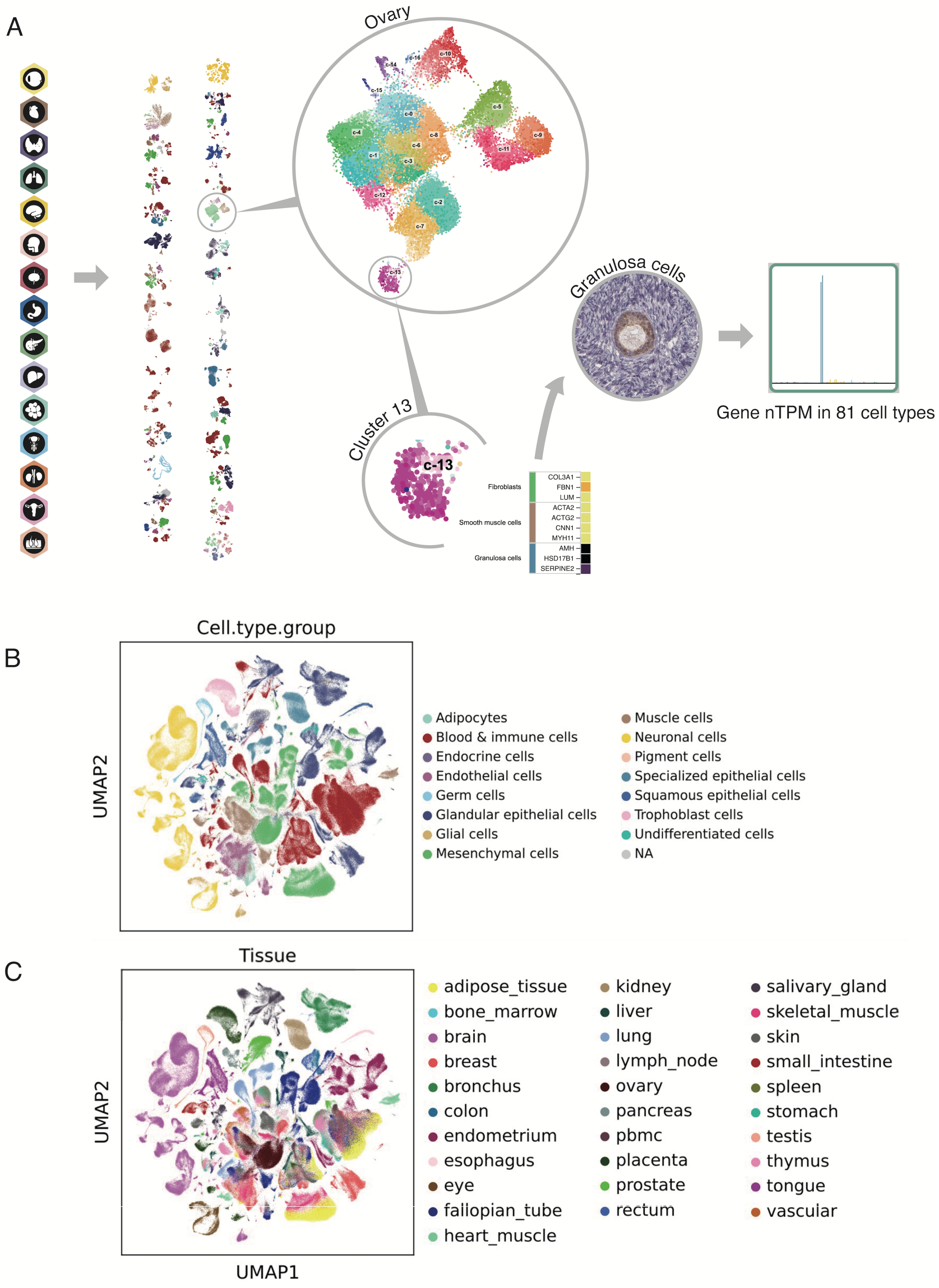

A
Ovary
c-10
c-16
c-14
c-15
c-0
c-5
c-4
c-8
c-9
c-6
c-1
c-11
c-3
c-12
c-2
c-7
c-13
Cluster 13
c-13
Fibroblasts
COL3A1
FBN1
LUM
Smooth muscle cells
ACTA2
ACTG2
CNN1
MYH11
Granulosa cells
AMH
HSD17B1
SERPINE2
Granulosa cells
Gene nTPM in 81 cell types
B
Cell.type.group
UMAP2
Adipocytes
Blood & immune cells
Endocrine cells
Endothelial cells
Germ cells
Glandular epithelial cells
Glial cells
Mesenchymal cells
Muscle cells
Neuronal cells
Pigment cells
Specialized epithelial cells
Squamous epithelial cells
Trophoblast cells
Undifferentiated cells
NA
C
Tissue
UMAP2
UMAP1
adipose_tissue
bone_marrow
brain
breast
bronchus
colon
endometrium
esophagus
eye
fallopian_tube
heart_muscle
kidney
liver
lung
lymph_node
ovary
pancreas
pbmc
placenta
prostate
rectum
salivary_gland
skeletal_muscle
skin
small_intestine
spleen
stomach
testis
thymus
tongue
vascular

Fig S2. Overview of the study, related to Figure 1.

(A) The workflow of HPA single cell section. Whole cell UMAP plot visualization on cell type groups (B) and tissues (C).

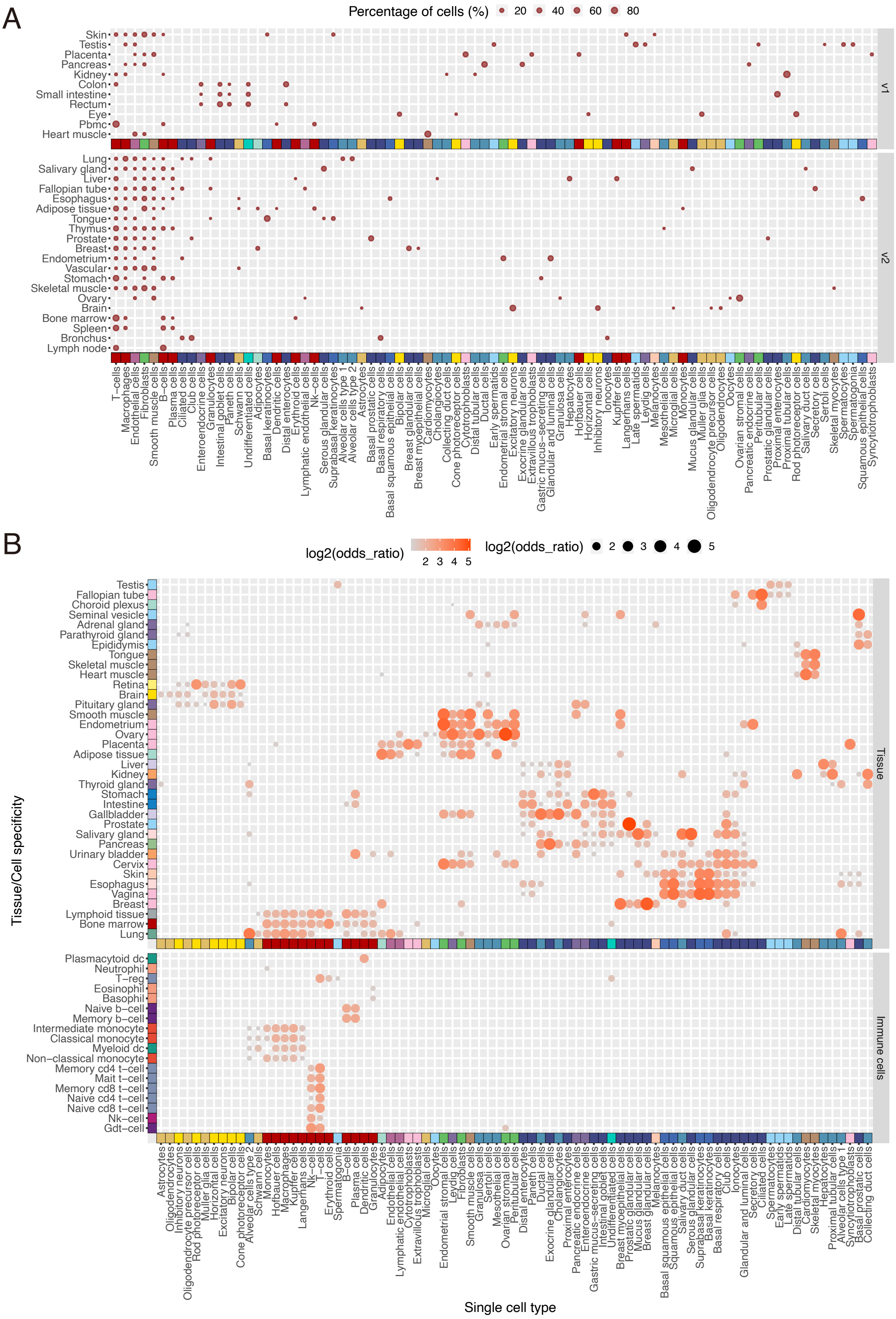
A
Percentage of cells (%) 20 40 60 80
Skin
Testis
Placenta
Pancreas
Kidney
Colon
Small intestine
Rectum
Eye
Pbmc
Heart muscle
v1
Lung
Salivary gland
Liver
Fallopian tube
Esophagus
Adipose tissue
Tongue
Thymus
Prostate
Breast
Endometrium
Vascular
Stomach
Skeletal muscle
Ovary
Brain
Bone marrow
Spleen
Bronchus
Lymph node
v2
T−cells
Macrophages
Endothelial cells
Fibroblasts
Smooth muscle cells
B−cells
Plasma cells
Ciliated cells
Club cells
Enteroendocrine cells
Granulocytes
Intestinal goblet cells
Paneth cells
Schwann cells
Undifferentiated cells
Adipocytes
Basal keratinocytes
Dendritic cells
Distal enterocytes
Erythroid cells
Lymphatic endothelial cells
Nk−cells
Serous glandular cells
Suprabasal keratinocytes
Alveolar cells type 1
Alveolar cells type 2
Astrocytes
Basal prostatic cells
Basal respiratory cells
Basal squamous epithelial cells
Bipolar cells
Breast glandular cells
Breast myoepithelial cells
Cardiomyocytes
Cholangiocytes
Collecting duct cells
Cone photoreceptor cells
Cytotrophoblasts
Distal tubular cells
Ductal cells
Early spermatids
Endometrial stromal cells
Excitatory neurons
Exocrine glandular cells
Extravillous trophoblasts
Gastric mucus−secreting cells
Glandular and luminal cells
Granulosa cells
Hepatocytes
Hofbauer cells
Horizontal cells
Inhibitory neurons
Ionocytes
Kupffer cells
Langerhans cells
Late spermatids
Leydig cells
Melanocytes
Mesothelial cells
Microglial cells
Monocytes
Mucus glandular cells
Muller glia cells
Oligodendrocyte precursor cells
Oligodendrocytes
Oocytes
Ovarian stromal cells
Pancreatic endocrine cells
Peritubular cells
Prostatic glandular cells
Proximal enterocytes
Proximal tubular cells
Rod photoreceptor cells
Salivary duct cells
Secretory cells
Sertoli cells
Skeletal myocytes
Spermatocytes
Spermatogonia
Squamous epithelial cells
Syncytiotrophoblasts
B
log2(odds_ratio) 2 3 4 5
log2(odds_ratio) 2 3 4 5
Tissue/Cell specificity
Testis
Fallopian tube
Choroid plexus
Seminal vesicle
Adrenal gland
Parathyroid gland
Epididymis
Tongue
Skeletal muscle
Heart muscle
Retina
Brain
Pituitary gland
Smooth muscle
Endometrium
Ovary
Placenta
Adipose tissue
Liver
Kidney
Thyroid gland
Stomach
Intestine
Gallbladder
Prostate
Salivary gland
Pancreas
Urinary bladder
Cervix
Skin
Esophagus
Vagina
Breast
Lymphoid tissue
Bone marrow
Lung
Tissue
Plasmacytoid dc
Neutrophil
T−reg
Eosinophil
Basophil
Naive b−cell
Memory b−cell
Intermediate monocyte
Classical monocyte
Myeloid dc
Non−classical monocyte
Memory cd4 t−cell
Mait t−cell
Memory cd8 t−cell
Naive cd4 t−cell
Naive cd8 t−cell
Nk−cell
Gdt−cell
Immune cells
Astrocytes
Oligodendrocytes
Inhibitory neurons
Oligodendrocyte precursor cells
Rod photoreceptor cells
Muller glia cells
Horizontal cells
Excitatory neurons
Bipolar cells
Cone photoreceptor cells
Alveolar cells type 2
Schwann cells
Monocytes
Hofbauer cells
Macrophages
Kupffer cells
Langerhans cells
Nk−cells
T−cells
Erythroid cells
Spermatogonia
B−cells
Plasma cells
Dendritic cells
Granulocytes
Adipocytes
Endothelial cells
Lymphatic endothelial cells
Cytotrophoblasts
Extravillous trophoblasts
Microglial cells
Oocytes
Endometrial stromal cells
Leydig cells
Fibroblasts
Smooth muscle cells
Granulosa cells
Sertoli cells
Mesothelial cells
Ovarian stromal cells
Peritubular cells
Distal enterocytes
Paneth cells
Ductal cells
Exocrine glandular cells
Cholangiocytes
Proximal enterocytes
Pancreatic endocrine cells
Enteroendocrine cells
Gastric mucus−secreting cells
Intestinal goblet cells
Undifferentiated cells
Breast myoepithelial cells
Prostatic glandular cells
Mucus glandular cells
Breast glandular cells
Melanocytes
Basal squamous epithelial cells
Squamous epithelial cells
Salivary duct cells
Serous glandular cells
Suprabasal keratinocytes
Basal keratinocytes
Basal respiratory cells
Club cells
Ionocytes
Glandular and luminal cells
Secretory cells
Ciliated cells
Spermatocytes
Early spermatids
Late spermatids
Distal tubular cells
Cardiomyocytes
Skeletal myocytes
Hepatocytes
Proximal tubular cells
Alveolar cells type 1
Syncytiotrophoblasts
Basal prostatic cells
Collecting duct cells
Single cell type

Fig S3. Overview of the 81 cell types, related to Figure 1 and Figure 4.

(A) The bubble plot shows the percentage of cells of 31 tissues in 81 cell types. (B) The bubble heatmap shows the enrichment of enriched genes of cell types in bulk and immune cell transcriptomics.

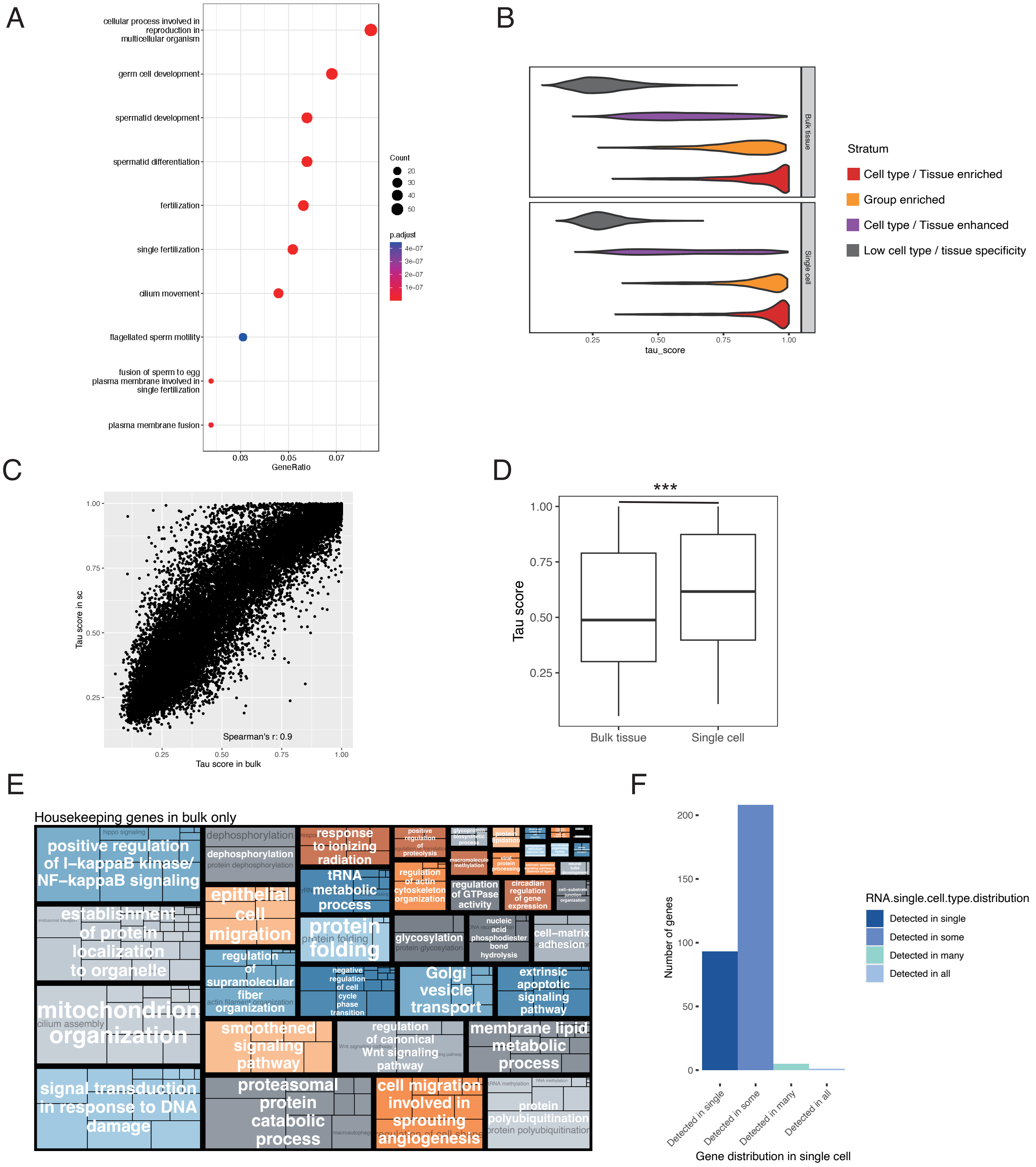

A
cellular process involved in reproduction in multicellular organism
germ cell development
spermatid development
spermatid differentiation
fertilization
single fertilization
cilium movement
flagellated sperm motility
fusion of sperm to egg plasma membrane involved in single fertilization
plasma membrane fusion
0.03
0.05
0.07
GeneRatio
Count
20
30
40
50
p.adjust
4e-07
3e-07
2e-07
1e-07
B
Bulk tissue
Single cell
0.25
0.50
0.75
1.00
tau_score
Stratum
Cell type / Tissue enriched
Group enriched
Cell type / Tissue enhanced
Low cell type / tissue specificity
C
1.00
0.75
0.50
0.25
Tau score in sc
Spearman's r: 0.9
0.25
0.50
0.75
1.00
Tau score in bulk
D
***
1.00
0.75
0.50
0.25
Tau score
Bulk tissue
Single cell
E
Housekeeping genes in bulk only
positive regulation of I−kappaB kinase/ NF−kappaB signaling
dephosphorylation
response to ionizing radiation
tRNA metabolic process
epithelial cell migration
protein folding
glycosylation
nucleic acid phosphodiester bond hydrolysis
cell−matrix adhesion
establishment of protein localization to organelle
regulation of supramolecular fiber organization
negative regulation of cell cycle phase transition
Golgi vesicle transport
extrinsic apoptotic signaling pathway
mitochondrion organization
cilium assembly
smoothened signaling pathway
regulation of canonical Wnt signaling pathway
membrane lipid metabolic process
signal transduction in response to DNA damage
proteasomal protein catabolic process
cell migration involved in sprouting angiogenesis
protein polyubiquitination
regulation of actin cytoskeleton organization
regulation of GTPase activity
circadian regulation of gene expression
positive regulation of proteolysis
macromolecule methylation
F
200
150
100
50
0
Number of genes
Detected in single
Detected in some
Detected in many
Detected in all
Gene distribution in single cell
RNA.single.cell.type.distribution
Detected in single
Detected in some
Detected in many
Detected in all

Fig S4. Gene classifications, related to Figure 2 and Figure 3.

(A) The bubble heatmap shows the enriched GO BP terms of spermatid enriched genes. (B) The violin plot shows the gene classification of genes and their corresponding tau score in single cell transcriptomics. The scatter plot (C) and the box plot (D) shows the similarity and differences of tau scores of consensus genes between single cell and bulk transcriptomics, respectively. (E) The tree map shows the enriched parent GO BP terms of bulk-only low specificity genes. (F) The bar plot shows the distribution of genes that only detected in single cell transcriptomics.
