# Supplementary material for: A resource for whole-body gene expression map of human tissues based on integration of single cell and bulk transcriptomics
Source: Genome Biol. 2025 Jun 3;26:152. doi: 10.1186/s13059-025-03616-4 (PMC12131445; doi:10.1186/s13059-025-03616-4)
Supplement: Supplementary file 3 — Additional File 3: Supplementary methods [file 13059_2025_3616_MOESM3_ESM.docx]

Supplementary methods

## **Key resource table**

| REAGENT or RESOURCE | SOURCE | IDENTIFIER |
| --- | --- | --- |
| Deposited Data | | |
| The Human Protein Atlas |  | <https://www.proteinatlas.org/> |
| Scripts | This paper | https://github.com/MengnanShi/HPA_SingleCellSection.git |
| Software and Algorithms | | |
| ggplot2 (v 3.4.0) | Wickham H et al.[1] | <https://cran.r-project.org/web/packages/ggplot2/index.html> |
| pheatmap (v1.0.12), | Raivo Kolde[2] | <https://cran.r-project.org/web/packages/pheatmap/index.html> |
| ggdendro (v0.1.23) | de Vries A et al.[3] | <https://cran.r-project.org/web/packages/ggdendro/index.html> |
| ggalluvial (v0.12.3), | Brunson JC et al.[4] | <https://cran.r-project.org/web/packages/ggalluvial/index.html> |
| gridExtra (v2.3) | Baptiste Auguie et al.[5] | <https://cran.r-project.org/web/packages/gridExtra/index.html> |
| ggpubr (v0.4.0), | Kassambara, A et al.[6] | <https://cran.r-project.org/web/packages/ggpubr/index.html> |
| cowplot (v1.1.1) | Claus O. Wilke[7] | <https://cran.r-project.org/web/packages/cowplot/index.html> |
| RColorBrewer (v1.1-3) | Erich Neuwirth[8] | <https://cran.r-project.org/web/packages/RColorBrewer/index.html> |
| circlize (v0.4.15) | Zuguang Gu[9] | <https://CRAN.R-project.org/package=circlize> |
| clusterProfiler (v4.2.2) | Wu T et al.[10] | <https://bioconductor.org/packages/release/bioc/html/clusterProfiler.html> |
| enrichplot (v1.14.2) | Guangchuang Yu et al.[11] | <https://bioconductor.org/packages/release/bioc/html/enrichplot.html> |
| ggsci (v2.9) | Nan Xiao[12] | <https://CRAN.R-project.org/package=ggsci> |
| polycor (v0.8-1) | John Fox[13] | <https://CRAN.R-project.org/package=polycor> |
| aplot (v0.1.8) | Guangchuang Yu[14] | <https://CRAN.R-project.org/package=aplot> |
| dendextend (v1.16.0) | Tal Galili et al.[15] | <https://CRAN.R-project.org/package=dendextend> |
| DWLS | Daphne Tsoucas et al.[16] |  |
| Cell Ranger (v6.1.2) | Zheng, G.X et al. | <https://www.10xgenomics.com/support/software/cell-ranger/latest/tutorials/cr-tutorial-in> |
| SRA Toolkit (v2.10.9) |  | <https://github.com/ncbi/sra-tools/wiki/01.-Downloading-SRA-Toolkit> |
| STAR (v2.7.9a) | Dobin, A. et al.[17] | <https://github.com/alexdobin/STAR> |
| Scanpy (v1.7.1) | Wolf, F. et al.[18] | <https://anaconda.org/conda-forge/scanpy> |
| pandas (v1.5.3) | The pandas development team[19] | <https://anaconda.org/conda-forge/pandas> |
| anndata (v0.8.0) | Isaac Virshup et al.[20] | <https://anaconda.org/conda-forge/anndata> |
| numpy (v1.23.5) | Harris, C.R. et al.[21] | <https://anaconda.org/conda-forge/numpy> |
| scrublet (v0.2.3) | Wolock, Samuel L et al.[22] | <https://anaconda.org/bioconda/scrublet> |
| Adobe illustrator 2021 |  | <https://www.adobe.com/se/> |

**Selection of single cell transcriptomic datasets**

The single cell RNA sequencing dataset is based on meta-analysis of literature on single cell RNA sequencing that include healthy human tissues. To avoid technical bias and to ensure that the single cell dataset can best represent the corresponding tissue, the following data selection criteria were applied: (1) Single cell transcriptomic datasets were limited to those based on the Chromium single cell gene expression platform from 10X Genomics (version 2 or 3); (2) Single cell RNA sequencing was performed on single cell suspension from tissues without pre-enrichment of cell types; (3) Only studies with >3,000 cells and 20 million read counts were included, (4) Only dataset whose pseudo-bulk transcriptomic expression profile is highly correlated with the transcriptomic expression profile of the corresponding HPA tissue bulk sample were included. It should be noted that exceptions were made for eye (~12.6 million reads), rectum (2,638 cells) and heart muscle (plate-based scRNA-seq) to include various cell types in the analysis.

In total, single cell transcriptomics data for 30 tissues and peripheral blood mononuclear cells (PBMCs) were analyzed. These datasets were respectively retrieved from the Single Cell Expression Atlas (https://www.ebi.ac.uk/gxa/sc/home), the Human Cell Atlas (https://www.humancellatlas.org), the Tabula Sapiens (https://tabula-sapiens-portal.ds.czbiohub.org), the Gene Expression Omnibus

(https://www.ncbi.nlm.nih.gov/geo), the Allen Brain Map (https://portal.brain-map.org), and the European Genome-phenome Archive (https://www.ebi.ac.uk/ega). The complete list of references is shown in **Table 1**.

**Clustering of single cell transcriptomics data**

For each of the single cell transcriptomics datasets, the raw sequencing data (Fastq files or SRA files) were downloaded from the corresponding repository database based on the accession number provided by the corresponding study in the available format. More in details, raw fastq files were downloaded directly for 25 tissues, including adipose tissue, bone marrow, breast, bronchus, endometrium, esophagus, eye, fallopian tube, heart muscle, lung, lymph node, ovary, pancreas, placenta, prostate, rectum, salivary gland, skeletal muscle, skin, small intestine, spleen, stomach, thymus, tongue, and vasculature. The fastq files of other 5 tissues and organs including colon, kidney, liver, PBMC and testis, were converted from SRA files using SRA Toolkit (v2.10.9). The quantified raw counting data was downloaded for brain specifically.

The single cell RNA-seq data processing followed the same pipeline as the HPA project. To quantify the transcript levels, the sequencing data were mapped to the human reference GRCh38.p13 cDNA, while datasets generated by the droplet-based 10X Genomics Chromium (10X) approach were processed by Cell Ranger (v6.1.2) [23], and datasets generated by the plate-based scRNA-seq were processed by STAR (v2.7.9a) [17]. Based on the annotation from Ensembl Archive Release 103 (from HPA v23, gene ensemble ID were mapped to Ensembl Archive Release 109), the transcript abundances were aggregated into gene level as read counts, and these count matrices from the same tissue were further aggregated into one matrix. This result in 31 count matrices for 31 tissues, respectively, with a total of 60,666 genes included for further analysis. The downstream analysis followed an in-house pipeline using Scanpy (v1.7.1) in Python 3.8.5 [18]. In the pipeline, the data were filtered using two criteria: a cell is considered as valid if at least 200 genes are detected, and a gene is considered as valid if it is expressed in at least 10% of the cells. Mitochondiral genes and ribosome genes cutoff were set based on different dataset (**Table S1**). For tissues containing more than 10,000 cells, 1000 cells were used as cutoff. Subsequently, the cell counts were normalized to have a total count per cell of 10,000. For each dataset, the valid cells were then clustered using Louvain clustering function within Single-Cell Analysis in Python (Scanpy)[18]. Default values of parameters were used in clustering. More in detail, the features of cells were projected into a PCA space with 50 components using UMAP, and a k-nearest neighbours (KNN) graph was generated. 15 neighbours were used in the network for Louvain.

**Single cell type annotation**

Each of the 557 different cell type clusters was manually annotated based on an extensive survey of well-known tissue- and cell type–specific markers, including both markers from the original publications and additional markers used in pathology diagnostics. For most single cell types, three marker genes were used, and for each cluster, one main cell type was chosen based on the overall expression pattern of all the marker genes. A reliability score (high, medium, low, very low) was assigned to each annotated cluster based on i) the level and specificity of the expression of the assigned main cell type marker genes in the cluster compared to other clusters, and ii) the degree of expression of marker genes specific to cell types other than the assigned main cell type. Each cell type was identified by three marker genes, and a high reliability score was given to clusters were a high expression was observed for all three cell type marker genes, in combination with very low expression of marker genes representing other cell types. Medium reliability corresponds to high expression of at least 2 cell type marker genes in combination with low expression of marker genes representing other cell types, while low reliability clusters had equally high expression of at least one marker gene representing another cell type, indicating that the cluster may contain more than one cell type. Finally, very low reliability cell type clusters had equally high expression of multiple cell type markers representing different cell types, indicating there was no clear majority of a certain cell type in the cluster. The most relevant markers (**Table S3**) are presented in a heatmap on the in the Single Cell Type section on each organ- and gene-specific page to clarify cluster annotation to visitors**.**

**Gene normalization and classification**

The expression data from single cell and bulk was aggregated from 557 single cell type clusters into 81 unique single cell types. The total read counts for all genes in each cluster was calculated by adding up the read counts of each gene in all cells belonging to the corresponding cluster. Finally, the read counts were scaled to transcripts per million protein-coding genes (pTPM) for each of the single cell clusters and then normalized (nTPM) using Trimmed mean of M values (TMM) to allow for between-cluster comparisons. The calculation of the nTPM matrix can be described as follows (formula *(1)*), where x represents the pTPM expression matrix, and i and j represents gene id and cluster id, respectively:

| $\hat{x}_{ij}=TMM\left( x_{ij}, reference={median\_column}_{i} \right);{median\_column}_{i}=median(x_{i,1},x_{i,2},\ldots,x_{i,n})$ | *(1)* |
| --- | --- |
|  |  |

To generate expression values per cell type, firstly, clusters were aggregated per cell type by calculating the weighted mean nTPM in all cells with the same cluster annotation within a tissue, as shown in formula *(2)*:

| $\hat{x}_{j}=\frac{\sum_{j=1}^{n} w_{j}x_{j}}{\sum_{j=1}^{n} w_{j}}$ | *(2)* |
| --- | --- |

where x is the vector of nTPM expression values of cluster j; n is the number of clusters that have the same cluster annotation within a tissue; w is the cell counts of cluster j. Then, the values for the same cell types in different tissues were mean averaged to a single aggregated value. Only clusters with medium and high reliability were included and clusters containing mixed cell types, Neutrophils and Platelets were excluded due to their low RNA content.

A genome-wide classification of the protein-coding genes regarding single cell type specificity has been performed using between-sample normalized data (nTPM). The genes were classified according to specificity into (i) cell type enriched genes with at least four-fold higher expression levels in one cell type as compared with any other analyzed cell type; (ii) group enriched genes with at least four-fold higher expression levels in a small number of cell types (2 to 10); (iii) cell type enhanced genes with expression levels at least four times higher in a single cell type when compared to the average expression level of that gene across all cell types; (iv) low cell type specificity genes with nTPM ≥ 1 in at least one cell type but not elevated in any cell type; (v) not detected genes with nTPM < 1 in all cell types; and (vi) not available genes due to the incompatible of Ensembl 103 and 109. Additionally, log10(nTPM + 1) transformed values were used to calculate the “tau” specificity score. Tau is defined [24] as formula *(3),* where x is vector of expression values across tissues:

| $\tau= \frac{\sum_{i=1}^{n} 1-\hat{x}_{i}}{n-1};\hat{x}_{i}=\frac{x_{i}}{\max_{0\leq i\leq n} x_{i}}$ | *(3)* |
| --- | --- |

**Deconvolution**

Bulk transcriptomic profile were obtained from 25 matched tissues in HPA, and then log2 scaled nTPM expression data were subjected to deconvolution using dampened weighted least squares (DWLS) [16]. To generate the signature matrix for deconvolution, log2 scaled nTPM expression data from 2109 cell-type enriched genes in 81 different cell types were employed.

The top 5 predicted cell types of the estimated results were extracted and visualized by bubble heatmap.

**Polyserial correlation**

In order to calculate the correlation between Tau score (continuous variable) and gene classifications (discrete variable), polyserial correlation was applied using the polyserial function from the R package polycor (v 0.8-1) with maximum-likelihood estimator [13]. Gene classifications were converted from character to ordinal variables as follows: "Cell type enriched" = 4, "Group enriched" = 3, "Cell type enhanced" = 2, and "Low cell type specificity" = 1.

**Quantification and statistical analysis**

Enriched pathways are considered to be significant if their Benjamini-Hochberg procedure (FDR) less than 0.05. To compare the tau scores of single cell dataset and bulk tissue dataset, independent samples t-test is applied. The statistical details of all analyses are described in the figure legends.

**Additional resources**

The Human Protein Atlas single cell type section: <https://www.proteinatlas.org/humanproteome/single+cell+type>

1. **ggplot2: Elegant Graphics for Data Analysis** [<https://ggplot2.tidyverse.org>]

2. **pheatmap: Pretty Heatmaps** [<https://CRAN.R-project.org/package=pheatmap>]

3. **ggdendro: Create Dendrograms and Tree Diagrams Using 'ggplot2'** [<https://andrie.github.io/ggdendro/>]

4. Brunson JC: **ggalluvial: Layered Grammar for Alluvial Plots.** *J Open Source Softw* 2020, **5**.

5. **gridExtra: Miscellaneous Functions for "Grid" Graphics** [<https://CRAN.R-project.org/package=gridExtra>]

6. **'ggplot2' Based Publication Ready Plots [R package ggpubr version 0.4.0]** [<https://cran.r-project.org/web/packages/ggpubr/index.html>]

7. **cowplot: Streamlined Plot Theme and Plot Annotations for 'ggplot2'** [<https://CRAN.R-project.org/package=cowplot>]

8. **RColorBrewer: ColorBrewer Palettes**

9. Gu Z, Gu L, Eils R, Schlesner M, Brors B: **circlize Implements and enhances circular visualization in R.** *Bioinformatics* 2014, **30:**2811-2812.

10. Wu T, Hu E, Xu S, Chen M, Guo P, Dai Z, Feng T, Zhou L, Tang W, Zhan L, et al: **clusterProfiler 4.0: A universal enrichment tool for interpreting omics data.** *Innovation (Camb)* 2021, **2:**100141.

11. **enrichplot: Visualization of Functional Enrichment Result** [<https://yulab-smu.top/biomedical-knowledge-mining-book/>]

12. **ggsci: Scientific Journal and Sci-Fi Themed Color Palettes for 'ggplot2'** [<https://nanx.me/ggsci/>, <https://github.com/nanxstats/ggsci>]

13. **polycor: Polychoric and Polyserial Correlations** [<https://CRAN.R-project.org/package=polycor>]

14. **aplot: Decorate a 'ggplot' with Associated Information** [<https://CRAN.R-project.org/package=aplot>]

15. Galili T: **dendextend: an R package for visualizing, adjusting and comparing trees of hierarchical clustering.** *Bioinformatics* 2015, **31:**3718-3720.

16. Tsoucas D, Dong R, Chen H, Zhu Q, Guo G, Yuan GC: **Accurate estimation of cell-type composition from gene expression data.** *Nat Commun* 2019, **10:**2975.

17. Dobin A, Davis CA, Schlesinger F, Drenkow J, Zaleski C, Jha S, Batut P, Chaisson M, Gingeras TR: **STAR: ultrafast universal RNA-seq aligner.** *Bioinformatics* 2013, **29:**15-21.

18. Wolf FA, Angerer P, Theis FJ: **SCANPY: large-scale single-cell gene expression data analysis.** *Genome Biol* 2018, **19:**15.

19. **pandas-dev/pandas: Pandas** [<https://doi.org/10.5281/zenodo.3509134>]

20. Virshup I, Rybakov S, Theis FJ, Angerer P, Wolf FA: **anndata: Annotated data.** *bioRxiv* 2021**:**2021.2012.2016.473007.

21. Harris CR, Millman KJ, van der Walt SJ, Gommers R, Virtanen P, Cournapeau D, Wieser E, Taylor J, Berg S, Smith NJ, et al: **Array programming with NumPy.** *Nature* 2020, **585:**357-362.

22. Wolock SL, Lopez R, Klein AM: **Scrublet: Computational Identification of Cell Doublets in Single-Cell Transcriptomic Data.** *Cell Syst* 2019, **8:**281-291 e289.

23. Zheng GX, Terry JM, Belgrader P, Ryvkin P, Bent ZW, Wilson R, Ziraldo SB, Wheeler TD, McDermott GP, Zhu J, et al: **Massively parallel digital transcriptional profiling of single cells.** *Nat Commun* 2017, **8:**14049.

24. Yanai I, Benjamin H, Shmoish M, Chalifa-Caspi V, Shklar M, Ophir R, Bar-Even A, Horn-Saban S, Safran M, Domany E, et al: **Genome-wide midrange transcription profiles reveal expression level relationships in human tissue specification.** *Bioinformatics* 2005, **21:**650-659.
